# Sequential preoperative hepatic vein embolization after portal vein embolization for extended left hepatectomy in colorectal liver metastases

**DOI:** 10.1186/1477-7819-11-134

**Published:** 2013-06-11

**Authors:** Gitonga Munene, Robyn D Parker, John Larrigan, Jason Wong, Francis Sutherland, Elijah Dixon

**Affiliations:** 1 Department of General Surgery, University of Tennessee Health Center, 1325 Eastmoreland Avenue, Suite 140, Memphis, TN 38104-7540, USA; 2 Department of Surgery and Surgical Oncology, University of Calgary, Foothills Medical Center, 1403, 29th Street NW, Calgary, AB T2N 2T9, Canada; 3 Department of Interventional Radiology, University of Calgary, Foothills Medical Center, 1403, 29th Street NW, Calgary, AB T2N 2T9, Canada

**Keywords:** Colorectal liver metastases, Hepatic vein embolization, Hepatectomy

## Abstract

**Background:**

The role of portal vein embolization to increase future liver remnant (FLR) is well-established in the treatment of colorectal liver metastases. However, the role of hepatic vein embolization is unclear.

**Case report:**

A patient with colorectal liver metastases received neoadjuvant chemotherapy prior to attempted resection. At the time of resection his tumor appeared to invade the left and middle hepatic vein, requiring an extended left hepatectomy including segments five and eight. Post-operatively, he underwent sequential left portal vein embolization followed by left hepatic vein embolization and finally, middle hepatic vein embolization. Hepatic vein embolization was performed to increase the FLR as well as to allow collateral drainage of the FLR to develop. A left trisectionectomy was then performed and no evidence of postoperative liver congestion or morbidity was found.

**Conclusion:**

Sequential portal vein embolization and hepatic vein embolization for extended left hepatectomy may be considered to increase FLR and may prevent right hepatic congestion after sacrificing the middle vein.

## Background

Curative hepatic resection is the most effective treatment of colorectal liver metastases. However, a large number of patients present with disease deemed to be unresectable. A multimodal approach which includes neoadjuvant chemotherapy and/or portal vein embolization has increased the resectability rates of patients with colorectal liver metastases [1]. Recently, hepatic vein embolization has been utilized to increase the resectability of patients with hepatobiliary malignancies to enable safe resection [2,3]. Both portal vein embolization and hepatic vein embolization have been shown to increase the future liver remnant. In addition to increasing future liver remnant (FLR), hepatic vein embolization may also reduce hepatic venous congestion by allowing collaterals to develop prior to resection. This report describes a case of preoperative sequential portal vein embolization followed by left and middle hepatic vein embolization for colorectal liver metastases.

## Case presentation

A 66 year-old male received a computed tomography (CT) scan of the abdomen one year after resection of a rectal cancer. His rectal cancer had been treated with low anterior resection and neoadjuvant chemotherapy, and pathology demonstrated a T3N1M0, moderately differentiated adenocarcinoma. He was referred to the hepatobiliary service for consultation. At presentation, he was asymptomatic. His past medical history was significant for hypertension. His only prior surgical procedure was low anterior resection for rectal cancer. On examination, he did not appear jaundiced; his abdominal examination was unremarkable and laboratory investigations were normal. His CT scan showed a single metastatic deposit in segment one of the liver. A positron emission tomography – computed tomography (PET/CT) scan was consistent with the CT with no extrahepatic disease found. At our institution, decisions regarding pre-surgery chemotherapy are made on a case-by-case basis. When the wait for surgery is likely to be beyond four weeks, usually patients will be offered chemotherapy. In this case, response to the various interventional radiology procedures was well beyond four weeks and for this reason chemotherapy was offered. A referral was made to medical oncologists for neoadjuvant chemotherapy and the patient received six cycles of FOLFOX. A postchemotherapy CT scan showed stable disease. Liver resection was attempted four weeks after the completion of his chemotherapy. A bilateral subcostal incision was made. After liver mobilization, it appeared that the tumor was larger than initially anticipated and had invaded past the middle hepatic vein and extended to the right of the middle vein, requiring a left trisectionectomy.

**Figure 1:**
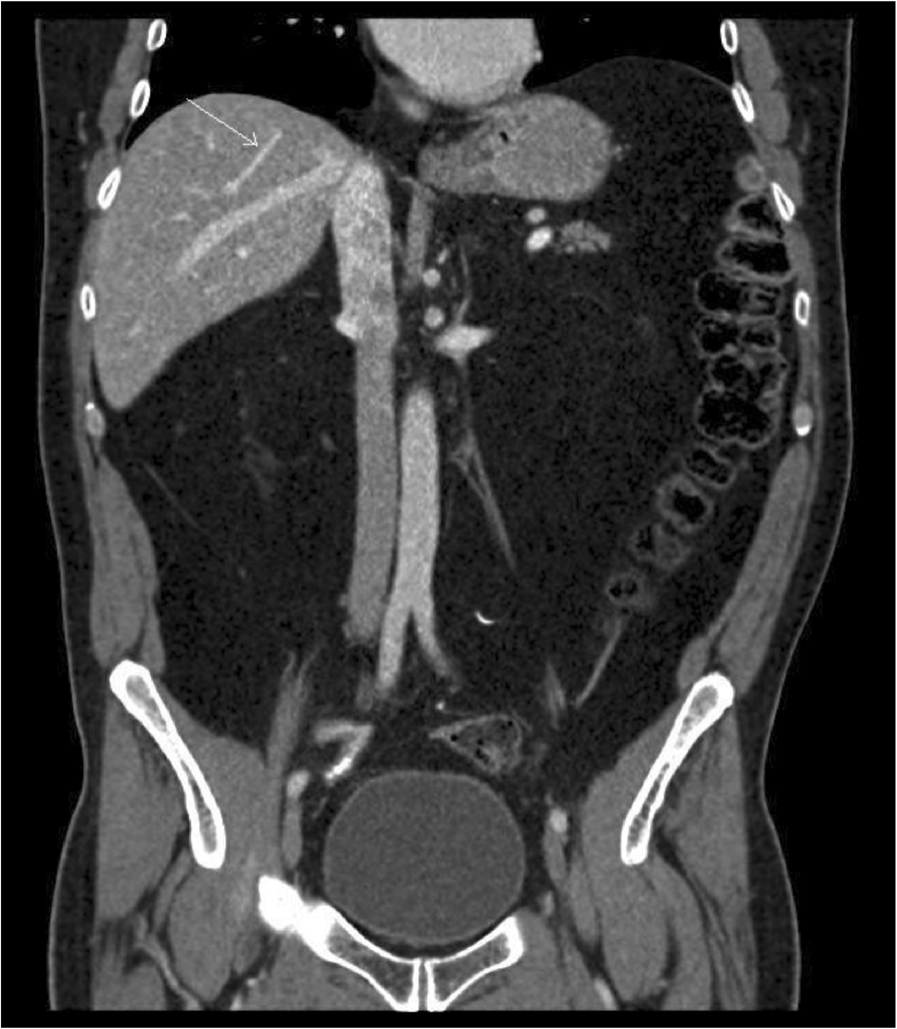
CT scan showing large middle vein tributary (arrow) draining the right liver.

**Figure 2:**
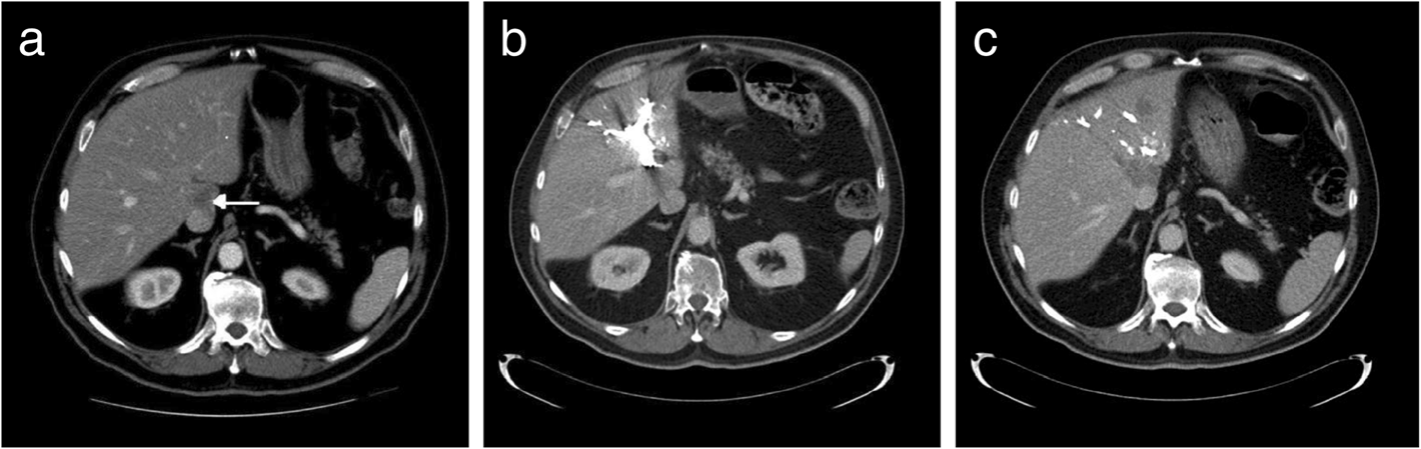
CT scans showing portal vein embolization and left and middle hepatic vein embolization a) CT scan showing metastatic lesion (arrow), b) CT scan after portal vein embolization, c) CT scan after left and middle hepatic vein embolization.

Intraoperative ultrasound (US) confirmed that an extended left hepatectomy would be required to achieve a R0 resection. A decision was made to abort the operation, given the concern that his liver appeared grossly steatotic; his posterior sector would be less than 25% of his total liver volume; and there was potential for hepatic congestion after middle vein ligation, especially with a prominent middle vein tributary draining the right segment (Figure 1). His postoperative course was unremarkable. Two weeks later he underwent left portal vein embolization with cyanoaccryalate lipidol without complications. Three weeks later he underwent left hepatic vein embolization. One week after left hepatic vein embolization, a middle hepatic vein embolization was performed in similar fashion without complication (Figure 2). Hepatic vein embolization was performed with an Amplatzer vascular plug (AGA Medical Corp.) via a right internal jugular vein access. Position of the plug was confirmed in the angiography suite using a multi-detector limited arc CT (XperCT, Philips Healthcare) with a guidewire within the targeted hepatic vein. Four weeks after middle hepatic vein embolization, an extended left hepatectomy was performed using the same incision. Intraoperatively, an atrophic left lobe was noted and a left extended hepatectomy with total vascular exclusion, five minutes preconditioning and an estimated blood loss (EBL) of 1,000 cc was performed. His postoperative course was uneventful and on postoperative day five, his international normalized ratio (INR) was 1.2 and total bilirubin was 52 μmol per liter. Pathology revealed three tumors in the resected specimen consistent with metastatic adenocarcinoma of colorectal origin, with negative microscopic margins and areas of necrosis together with patchy steatosis and stage two fibrosis.

## Discussion

This is the first case of sequential portal vein embolization and combined left and middle hepatic vein embolization reported in the literature. Currently, the mortality associated with liver resection ranges from 0 to 5% with a morbidity ranging from 10 to 20% [4,5]. The morbidity and mortality of extended left hepatectomy is higher than that of major resections with a reported mortality of 0% to 11.9 % and morbidity of 53% [6]. The most common cause of mortality is abdominal sepsis and postresectional liver failure secondary to insufficient FLR. Consequently, different techniques have been used to increase FLR. In addition to ensuring an adequate FLR, the risk of postoperative hepatic congestion in our patient was significant given the patient’s right-dominant middle hepatic vein.

Portal vein embolization (PVE) has been shown to increase FLR between 7% to 27% of the total liver volume (TLV) or 20 to 46% beyond pre-PVE FLR two to eight weeks after PVE, and is therefore used to increase FLR in patients deemed to have a small remnant liver [1,4,7]. PVE has been shown to increase resectability rate in up to 7% of patients with a benefit in postoperative outcomes. The sufficient FLR required in a normal liver is > 20% of the total liver volume; in patients with impaired liver function this is believed to be > 40% [5].

Chemotherapy has been associated with increased risk of liver injury: fluorouracil (5-FU) has been associated with steatosis; oxaliplatin-based regimens have been associated with vascular injuries; and irinotecan-based regimens have been associated with steatosis and steatohepatitis. The exact consequence of these histopathological lesions is unclear but patients with steatohepatitis have been shown to have higher incidences of posthepatectomy complications [8]. The regenerative ability of chemotherapy injured livers has been shown to be unimpaired following portal vein embolization [9].

PVE has been utilized in extended right hepatectomies, has been found to be safe and can increase the remnant liver [10]. In this case, the patient’s liver appeared to be grossly steatotic and the ideal FLR required for a safe resection was unclear. In this case, on volumetric analyses the posterior section appeared to be < 25% of total liver volume and the left portal vein was embolized with an increase in FLR to 30%.

PVE is not without risks; the greatest concern is for rapid tumor growth due to the increased production of growth stimulants, presumably in response to PVE. Animal models and one human study in humans have produced inconclusive data [11]. After PVE, careful monitoring of tumor response is warranted, and some have recommended concurrent chemotherapy after PVE to control for the deleterious effects PVE may have on tumor growth [8,12].

The role of hepatic vein embolization in improving resectability rates and the safety of extended resections of patients with FLR < 20% after PVE is unclear. Hwang *et al*. recently showed, in a study of 12 patients who underwent hepatic resection for hepatobiliary malignancy, that sequential right hepatic vein embolization after right PVE had an incremental effect on the FLR. Immunohistochemistry showed increased apopotosis after hepatic vein embolization following PVE, suggesting that hepatic vein embolization induced a more severe injury. Hepatic vein embolization was shown to be safe and did not appear to increase blood liver enzymes any more than did portal vein embolization [2]. The study only included patients who underwent right-sided resections and therefore, only patients who underwent right hepatic vein embolization [2].

This report is the first to document the use of left and middle vein embolization in an attempt to increase FLR. In this case, after PVE, the FLR increased to > 30%, but after hepatic vein embolization no additional increase was documented on volumetric analysis. There was a transient increase in liver enzymes after portal vein and hepatic vein embolization which normalized prior to the curative resection, and no complications were associated with either procedure.

Venous congestion associated with sacrificing the middle hepatic vein has been associated with physiological impairment, functional small-for-size syndrome, loss of the graft and death of living liver transplant donors [13-18]. Management of the middle hepatic vein remains a controversial topic, especially for right graft living donor liver transplants. Using CT volumetric analysis, many centers have devised algorithms that attempt to predict the risk of congestion associated with middle hepatic vein harvest [13,18,19]. Some centers have advocated the utilization of vein reconstruction to prevent congestion [16]. Congestion leads to necrosis of the marginal zones of the remnant liver and may lead to increased biliary complications and potentially infection. Scatton *et al*. showed that hepatic congestion was associated with impaired liver function and volumetric regeneration. Even though the clinical consequences of congestion were moderate in the healthy donor population, this effect may be severe in patients after extended resection with small remnants and diseased livers [20].

The role of hepatic congestion in decreasing the remnant liver and increasing morbidity and mortality after extended resections has been documented in a number of studies [21-24]. Lang *et al*. found that, when using computer-assisted risk analysis to plan for major hepatectomies, the amount of devascularized liver tissue was greater than 20% and up to 43% of that anticipated by 2D CT. Most of the reduction in vascularized liver tissue resulted from hepatic congestion. In particular, for patients who were to undergo extended left hepatectomies, a large portion of segment six drained into the middle hepatic vein. Based on their preoperative findings, the surgical team involved planned vein reconstructions for these patients after intraoperative confirmation of hepatic congestion.

Multiple studies have reported on their experience of venous reconstruction to prevent hepatic congestion, with good outcomes. Venous reconstruction increases the complexity of the operation and is associated with complications [25]. We hypothesize that an alternative to venous reconstruction could be preoperative hepatic vein embolization to increase collateralization gradually so as to decrease early hepatic congestion. Our patient appeared to be at high risk of congestion given his large accessory vein draining from his right liver into the middle hepatic vein. Using an animal model, Ku *et al*. showed that preoperative hepatic vein embolization resulted in increased collateralization of interlobar and interlobular collaterals in two weeks. In the only published case report to document an increase in venous drainage after hepatic vein embolization, a right hepatic vein embolization was done to enable an inferior right hepatic vein-preserving left hepatic trisectionectomy with combined resection of the distal right hepatic vein without reconstruction. Preoperative hepatic vein embolization increased drainage via the inferior right hepatic vein, allowing a safe resection without congestion [22].

Preoperative studies can now identify patients with middle hepatic vein-dominant right livers, and a study to evaluate the benefits of preoperative hepatic embolization prior to resection in these groups of patients is warranted because it may decrease postoperative hepatic congestion, and may preclude the need for venous reconstruction. Hepatic vein embolization is associated with the risk of dislodgement of the embolic material because the hepatic vein is retrograde and the vein is larger distally than proximally.

## Conclusion

In conclusion, we have presented a case where a combination of neoadjuvant chemotherapy, sequential preoperative portal vein embolization and hepatic embolization enabled a safe left trisectionectomy with excellent outcome. The role of hepatic vein embolization to increase future liver remnant and to decrease postoperative hepatic congestion in well-selected patients warrants further study.

## Consent

Written informed consent was obtained from the patient for publication of this case report and accompanying images. A copy of the written consent is available for review by the editor-in-chief of this journal.

## Abbreviations

CT: Computed tomography; EBL: Estimated blood loss; FLR: Future liver remnant; INR: International normalized ratio; PET/CT: Positron emission tomography – computed tomography; PVE: Portal vein emolization; TLV: Total liver volume; US: Ultrasound.

## Competing interests

The authors declare that they have no competing interests.

## Authors’ contributions

GM, FS and ED participated in the surgical care of the patient. JL and JW were responsible for the interventional radiology, and all authors made major contributions to writing the manuscript. All authors read and approved the final manuscript.
